# Identification of SNPs in a nonmodel macrofungus (*Lepista nuda*, Basidiomycota) through RAD sequencing

**DOI:** 10.1186/s40064-016-3459-8

**Published:** 2016-10-13

**Authors:** Fei Ye, Xiao-Dan Yu, Qing Wang, Peng Zhao

**Affiliations:** 1 College of Biological Science and Technology, Shenyang Agricultural University, Shenyang, 110866 Liaoning People’s Republic of China; 2 College of Jilin Agricultural Science and Technology, Jilin, People’s Republic of China; 3 Provincial Key Laboratory of Forest Protection, Liaoning Academy of Forestry, Shenyang, Liaoning People’s Republic of China; 4 Key Laboratory of Shandong Province for Edible Mushroom Technology, Institute of Mycological Science and Technology, Ludong University, Yantai, Shandong People’s Republic of China

**Keywords:** Agaricales, Fungi, Single-nucleotide polymorphisms, Restriction-site associated DNA

## Abstract

*Lepista nuda* is a wild edible fungus that is valued for its odor and taste. Recent studies identified intraspecific morphological and genetic differences in *L. nuda*. Although single-nucleotide polymorphisms (SNPs) are useful for revealing intraspecific differences, the traditional methods used for investigating SNPs are time consuming and expensive, and they only locate a limited number of SNPs. This study used a “restriction-site associated DNA” (RAD) method combined with high throughput sequencing to efficiently identify a large number of SNPs in two samples of *L. nuda*. A total of 7 and 9 billion bp of raw data were obtained from the two collections. A total of 712 SNPs were found. These SNPs will be useful for the further analysis of the genetic variation within *L. nuda*. The study also confirms that the RAD method can be used to identify SNPs in a nonmodel macrofungus for which a reference genome is unavailable.

## Background

*Lepista nuda* (Bull.) Cooke, which is in the Agaricales (Basidiomycota) (Kirk et al. 2008), is a wild, edible mushroom that is common in many parts of the world (Singer 1986). This fungus is popular because of its taste, smell, and nutritional qualities and is considered edible in Europe (Singer 1986), China (Dai et al. 2010), and elsewhere. Consequently, the understanding and conserving of natural populations of *L. nuda* has attracted significant attention from mushroom collectors, government agencies, and conservation groups.

Descriptions of *L. nuda* morphology vary considerably. The pileus of *L. nuda*, for example, was described as grey brown or russet brown by some researchers but was described as purple brown by the other (Bon 1987; Hansen and Knudsen 1992; Mao 2000). Molecular data for the species also reveal substantial intraspecific differences. Thus, the analysis of ITS sequences showed that two collections of *L*. *nuda* did not group together (Moncalvo et al. 2002). Using amplification polymorphism fragments (CAPS) and random amplified polymorphisms (RAPD), the results showed that the *L*. *nuda* from Greece and the United States differ from the *L. nuda* in France and Australia (Stott et al. 2005). To date, few reports have considered the intraspecific molecular variability of *L. nuda*, probably because the molecular methods available are time consuming and costly. Therefore, rapid and inexpensive methods are needed to clarify the morphological and genetic variation within *L. nuda*.

Single-nucleotide polymorphisms (SNPs) refer to variations in a single base pair of a DNA sequence. SNPs, which can function as useful markers for the study on population genetic, consist of unlinked loci that occur throughout the genome and that have relatively low mutation rates (Brumbfield et al. 2003). Generally, SNPs were developed mainly by DNA sequencing. To identify SNPs in *Tricholoma matsutake*, a genomic library was constructed and 73,065 bp were sequenced from random clones. Special primers from 20 sequenced fragments were then designed to amplify and analyzed more than 10,428 bp sequences from the two strains. Finally, a total of 178 SNPs were developed (Xu et al. 2007). There were only four SNPs from seven *Armillaria cepistipes* isolates by sequencing the regions of ten single-copy protein-coding homologues and the housekeeping gene EF1-α (Heinzelmann et al. 2012). Therefore, the traditional method for detecting SNPs is time consuming and expensive and detects only a limited numbers of SNPs.

An efficient method for identifying SNP loci combines “restriction-site associated DNA” (RAD) with high throughput sequencing (Miller et al. 2007; van Tassell et al. 2008). The advantages of this method include: (1) the number of SNPs identified is ten-times greater than with the traditional technology; (2) the data utilization rate is high, and the cost of sequencing is relatively low; (3) the time and work required are less than with the traditional method; and (4) the method can be used for species that lack a reference genome. RAD techniques have been widely used to find SNP loci in animals and plants (Bourgeois et al. 2013; Lamer et al. 2014; Yu et al. 2015; Wang et al. 2013; Zhao et al. 2014; Xiao et al. 2015). For fungi, the RAD method has been used for *Laccaria bicolor* and for the plant-pathogenic fungi *Pyrenophora teres* and *Sphaerulina musiva* (Wilson et al. 2015; Leboldus et al. 2015). In this study, the RAD method was combined with Illumina sequencing to discover the SNPs in *L. nuda*. These SNPs could be used for further research concerning the population genetics within *L. nuda*.

## Results and discussion

### Sample identification

Two ITS sequences from the dried basidiomata of the two *L. nuda* specimens (HMAS 254481 and HMAS 254482) were obtained in this study. The sequences were submitted to GenBank: the GenBank accession numbers are KU215618 and KU215619. To assess the taxonomic status of these specimens, the ITS sequences obtained from this study were compared with the sequences in GenBank by a BLAST database search (Altschul et al. 1997). The results showed >99 % identity between the sequences obtained from this study and the sequences named “*L. nuda*” in GenBank. Given these molecular characteristics and the morphological characteristics of the specimens, we conclude that the two specimens were *L. nuda*.

### Base calling

**Fig. 1:**
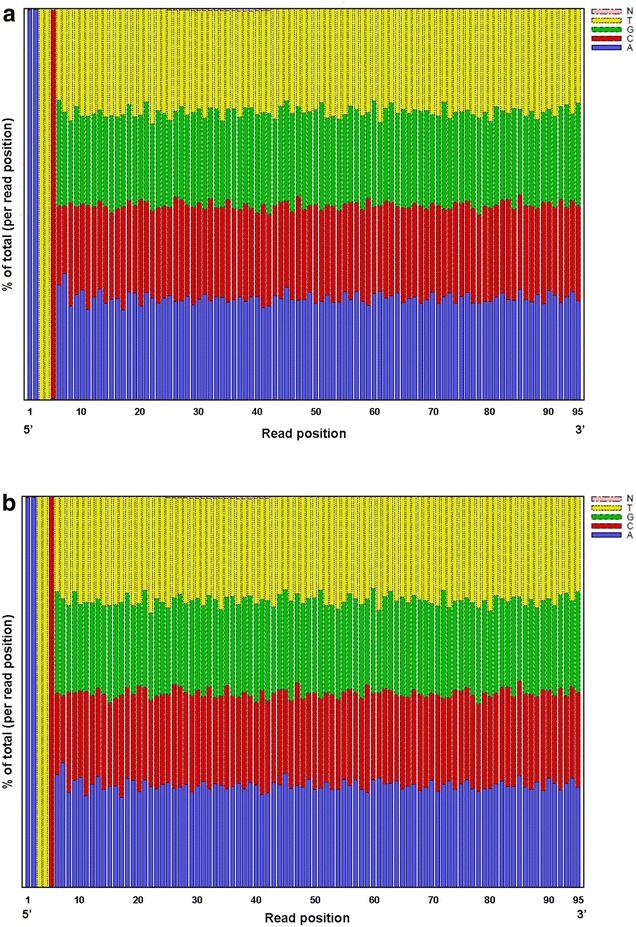
**a**, **b** The distribution of bases in two *L. nuda* collections. The first five bases are the restriction enzymes loci. **a** HMAS 254481, **b** HMAS 254482

**Fig. 2:**
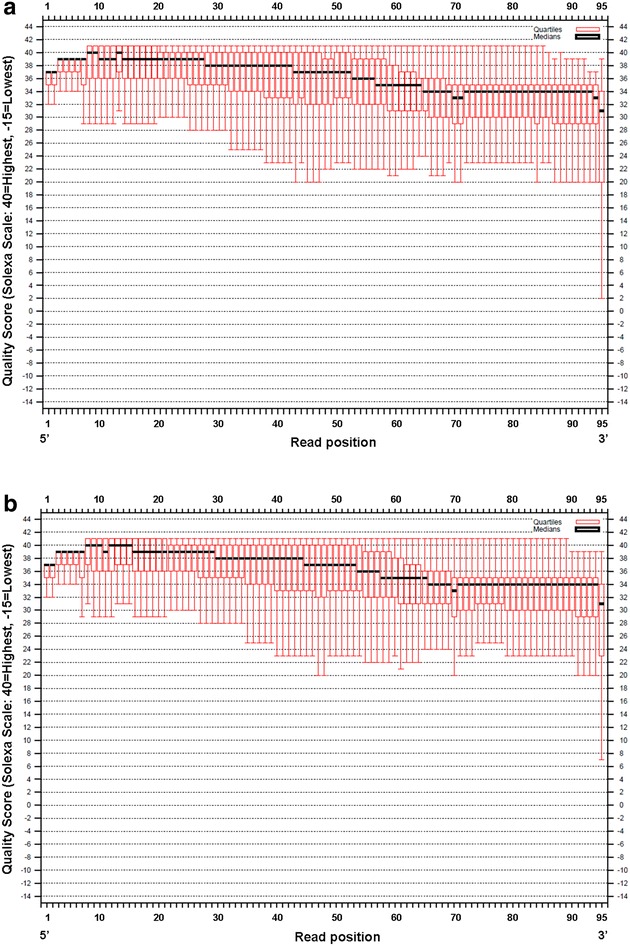
**a**, **b** The distribution of base quality in the two *L. nuda* collections. The *red vertical line* represents the base quality of reads; the *red vertical square* represents the quartile of the base quality; the *thick black line* represents the median of the quartile. **a** HMAS 254481, **b** HMAS 254482

In this study, the RAD method was used to find SNPs in *L. nuda*. The distribution of bases is presented in Fig. 1, and the distribution of base quality is presented in Fig. 2. The base composition was similar among all of the reads, and the percentage of N was very low (Fig. 1). The base quality, which reflects the error rate of sequencing, was high for both samples (Fig. 2). Quality declines during sequencing as the activity of enzymes and the amount of reagent decline. Because the activity of enzymes and the amount of reagent decline during sequencing, quality declines once a certain sequence length is attained (Wang et al. 2014). The base quality is also affected by the sequencing machine, the reagent, and the samples used. Statistical analysis indicated that the Q20 (indicating a 1 % sequencing error) of each sample was >92 %, and that the Q30 (indicating a 0.1 % sequencing error) of each sample was >84 % (Table 1). The mean GC content of the sequence in the two populations was about 46 %. A total of 7 billion bp of raw data was obtained from one sample and 9 billion from the other. Nine million or eight million clean reads were obtained by trimming the reads in the poor quality (quality value <Q20 and N > 10 %). The clean data was calculated by all of nucleotides of the clean reads without the adapter parts. The same reads cluster into a RAD-tag. There were about 170,000 RAD-tags in one sample and 140,000 in the other. The average depth was about 45× for one sample and 48× for the other. The overall sequencing depth in the current study was high. The redundancy of the two samples was relatively high (Table 1). There were two possible reasons why the redundancy was high. One possible reason was that all the reads were sequenced in forward direction. The other reason was that the size of genome of *L. nuda* is relatively small (about 70 Mb).

**Table 1:** Statistics for reads of two *L. nuda* samples

Collections	HMAS 254481	HMAS 254482
Read length	95 SE	95 SE
Raw data	913,113,400	777,371,035
Clean data	910,348,330	775,132,075
Number of clean reads	9,582,614	8,159,285
Number of RAD-tags	176,688	141,777
Average depth	45.8628	48.8587
Q20 (%)	92.92	93.38
Q30 (%)	83.64	84.49
GC content (%)	46.1	45.87
Redundancy (%)	26.8	22.1

*SE* single end

### SNP calling

**Fig. 3:**
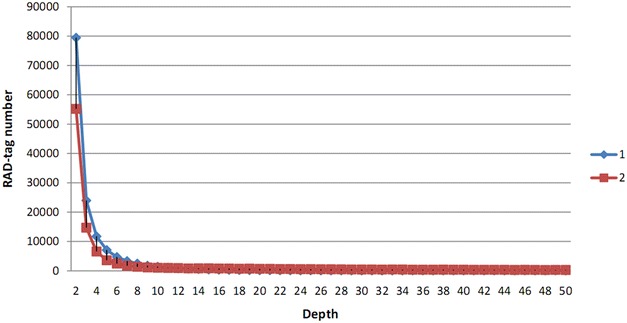
RAD-tag depth distribution in two *L. nuda* collections. *1* HMAS 254481, *2* HMAS 254482

The RAD-tag depth distribution is shown in Fig. 3. In order to discharging the sequencing errors, the SNPs <6× were removed. A total of 712 SNPs were identified from the RAD-tags of the two samples. The SNPs distribution in the depth of reads is listed in Table 2. The numbers of SNPs were more than the numbers of SNPs obtained from the studies by the traditional methods (Xu et al. 2007; Heinzelmann et al. 2012). Although Wilson et al. (2015) obtained a high number of SNPs (17,854) from *L. bicolor* samples using the RAD method, the samples included both ingroup and outgroup specimens. Therefore, the number of informative SNP markers obtained for *L. bicolor* ranged from about 322 to 1000. In this paper, 712 SNP loci were obtained from two *L. nuda* samples using the RAD method. This number of SNP loci is sufficient to support further study of the genetic variation of *L. nuda*. The results of this study support that the RAD method was useful for identifying SNP loci in species for which genomic information is lacking.

**Table 2:** The correspondence between the depth of reads and the proportion of SNPs

The depth of reads (×)	The proportion of SNPs (%)
6–10	4.2
10–20	14.5
20–30	13.7
30–40	16.9
40–50	17.4
Above 50	33.3

## Conclusions

This study used RAD method combined with high throughput sequencing to identify a total of 712 SNPs in two samples of *L. nuda*. These SNPs will be used to examine the population genetics of *L. nuda*. This paper also confirms that the RAD method can be used to identify SNPs in a nonmodel macrofungus for which a reference genome is unavailable. Furthermore, the SNPs could provide the theoretical support for further research about cultivation and breeding of *L. nuda*. It is important for the protection of resources and the conservation of the population genetics of *L. nuda* in the field.

## Methods

### Sample identification

Fungi used in this study were collected from Tianzhu Mountain, Shenyang City, Liaoning Province and Muleng town, Mudanjiang City, Heilongjiang Province of China (The locations are public areas. Therefore, there are no specific permissions were required for the locations. The authors confirm that the field studies did not involve endangered or protected species.). The specimens were dried with an electric air-ventilation drier and were deposited in the Mycological Herbarium of the Chinese Academy of Sciences (HMAS) with accession numbers of HMAS 254481 and HMAS 254482. Genomic DNA was extracted from the dried blocks of tissue of the herbarium specimens (Table 3) using the Plant DNA Extraction Kit (Sunbiotech Co., Ltd., Beijing, China) and following the manufacturer’s instructions. The crude DNA extracts were used as templates for PCR. Primers ITS5/ITS4 (GGAAGTAAAAGTCGTAACAAGG/TCCTCCGCTTATTGATATGC) were used for amplification of the ITS region including ITS1, 5.8S, and ITS2 (White et al. 1990). Reaction mixtures and PCR conditions were as described by the previous study (Yu et al. 2014). At the same time, the 16S region was amplified by the primers 27F/1492R (AGAGTTTGATCMTGGCTCAG/TACGGYTACCTTGTTACGACTT) for confirming the absence of non-fungal DNA (Lane 1991). The PCR products were checked on a 1 % agarose gel and visualized by staining with ethidium bromide. Sequencing was performed on an ABI Prism^®^ 3730 Genetic Analyzer (PE Applied Biosystems, Foster, CA, USA). Nucleotide sequences of the ITS regions that were amplified from the collections (HMAS 254481 and HMAS 254482) were aligned with the sequences of *L. nuda* ITS regions retrieved from GenBank using BioEdit 5.0.6 (Hall 1999) and Clustal X (Thompson et al. 1997).

**Table 3:** Collections of *L. nuda* used for the analysis

Taxa	Voucher collections	Origin	GenBank accession no.
*L. nuda*	HMAS 254481	Tianzhu Mountain, Shenyang City Liaoning Province, China	KU215618
*L. nuda*	HMAS 254482	Muleng Town, Mudanjiang City Heilongjiang Province, China	KU215619

### Creation and sequencing of the RAD library

For construction of the RAD library, the DNA from the two samples was pooled. The sample indexing and pooling were followed by the published method (Baird et al. 2008). Because a reference genome for *L. nuda* is unavailable, the genomic DNA was first digested with *Eco*RI (NEB Company). The digestion was performed at 37 °C overnight; digestion ended with a 20-min deactivation step at 65 °C followed by cooling to 4 °C and the enzyme-digested product was pooled. The fragments of digested DNA were connected to the P1 adapter using T4 DNA ligase (NEB company). The sequences of the P1 adapter include the *Eco*RI restriction site, a reverse amplification site, and the Illumina sequencing primer sites. Subsequently, the fragments were physically broken using a Covaris ultrasonicator with 200 bursts of 90 s each on high power, after which the resulting fragments were confirmed to be 300–500 bp in length by agarose gel electrophoresis. Then, a P2 adapter was connected to the pieces (300–500 bp). The sequences of the P2 adapter include the forward amplification and Illumina sequencing primer sites. After PCR amplification, only the fragments that included the P1 and P2 adapters were screened. Single-end (101 bp, including 6 bp for barcode) sequencing was performed using the Illumina HiSeq2000 in a total throughput of 16 lanes (Shanghai Majorbio Bio-pharm Technology Co., Ltd.).

### SNP calling

A total of 913,113,400 bp of raw data was obtained from one of the samples, and a total of 777,371,035 bp of raw data was obtained from the other. After processing, totals of 910,348,330 and 775,132,075 bp of clean data were derived from the processed raw data. The process was completed by SeqPrep and included removing the adapter parts, trimming the nucleotides that had a quality value <Q20, eliminating those reads in which N was >10 %. We distinguished the trimmed reads of the two samples according to the barcode, and the trimmed reads were clustered into read tags (hereafter referred to as RAD-tags) by sequence similarity using ustacks (Catchen et al. 2011) to produce unique candidate alleles for each RAD locus. A maximum base-pair mismatch of two was allowed in this step. RAD-tags were then collapsed into clusters using ustacks under default parameters for SNP calling.

## Abbreviations

CAPS: amplification polymorphism fragments
EF1-α: elongation factors 1-α
HMAS: the Mycological Herbarium of the Chinese Academy of Sciences
RAD: restriction-site associated DNA
RAPD: random amplified polymorphisms
SNPs: single-nucleotide polymorphisms
